# The effect of hyoscine n- butylbromide on labor progress: A systematic review

**DOI:** 10.1186/s12884-020-2832-3

**Published:** 2020-05-13

**Authors:** Zaynab Mohaghegh, Parvin Abedi, Shahla Faal, Shayesteh Jahanfar, Alayna Surdock, Foruzan Sharifipour, Maryam Zahedian

**Affiliations:** 1 Midwifery Department, Ahvaz Jundishapur University of Medical Sciences, Ahvaz, Iran; 2 Midwifery Department, Menopause Andropause Research Center, Ahvaz Jundishapur University of Medical Sciences, Ahvaz, Iran; 3 Department of Midwifery, Islamic Azad University, Marand Branch, Marand, Iran; 4 School of Health Sciences-MPH Program Health Professions Building 2212, Central Michigan University, Mount Pleasant, USA; 5 Central Michigan University (CMU), Mount Pleasant, USA; 6 Nursing and Midwifery School, Ahvaz Jundishapur University of Medical Science, Ahvaz, Iran

**Keywords:** Hyoscine n-butylbromide, First stage, Labor, Duration, Augmentation, Progress

## Abstract

**Background:**

The objective of this systematic review and meta-analysis was to assess the effectiveness of hyoscine n-butylbromide in labor progress.

**Methods:**

The databases including PubMed, the Cochrane Library, Science-Direct, Scopus and Web of Science were searched for studies published up to December 2019. Articles that published as randomized controlled trials (RCTs), and full-text articles published in English or other languages were included and participants were primi or multigravida women who were in active phase of labor. The intervention included HBB compared to placebo (normal saline) that was used during active phase of labor. Pooled estimates were measured using the fixed or random effect model, while the overall effect was reported in a mean difference (MD). All data were analyzed using Review Manager 5.3.

**Results:**

Twenty studies involving 3108 women were included in meta-analysis. Based on subgroup analysis by parity, use of HBB significantly reduced the duration of the first stage of labor in primigravida women (MD = − 57.73; 95% CI: [− 61.48, − 53.60]) and in multigravida women (MD = − 90.74; 95% CI: [− 97.24, − 84.24]). Administering HBB could reduce the second stages of labor in primigravidas and multigravidas about 6 min and 4 min respectively. Also, HBB reduced the duration of the third stage of labor in multigravidas about 3 min. APGAR score at one and 5 min after birth was not affected. The main maternal adverse effect was tachycardia and dry mouth. Labor duration in studies in which the participants were primi-and multigravida was not presented based on separate parities except for four papers, and the route of HBB administration was not the same across all studies.

**Conclusions:**

Although, the effect of HBB was minimal when multigravidas and primigravidas women were considered together, the HBB was clinically effective in primigravida and multigravida women for shortening the first and the second stages of labor. Also, HBB could reduce the length of the third stage of labor in multigravidas.

## Background

Improving maternal health and decreasing maternal mortality is one of the main concerns around the globe [1]. Everyday, about 830 women die from pregnancy or childbirth-related complications around the world. In 2015, it was estimated that, roughly 303,000 women died during or following pregnancy and childbirth. Almost all of these deaths occurred in low-income countries, and were mostly preventable [2]. Between 1990 and 2015, the global maternal mortality ratio (the number of maternal deaths per 100,000 live births) declined by only 2.3% per year. However, a significant decline of maternal mortality was observed from 2000 onwards. Reduction of the global maternal mortality ratio to less than 70 per 100,000 births, with no country having a maternal mortality rate of more than twice the global average is one of the objectives of the Sustainable Development Goals by the year 2030 [3].

Duration of labor is one the effective factors in maternal and fetal outcomes [4]. Prolonged labor can lead to increased maternal and neonatal morbidity and mortality such as rupture of the uterus, postpartum hemorrhage, puerperal sepsis, and maternal death [5]. Prolonged labor may be due to maternal age, pre mature rapture of membrane, epidural analgesia and the secretion of high levels of maternal stress hormones, but it remains unknown in most cases [6] .The mean duration of the first- and second stages of labor is approximately nine and 6 hours in nulliparous and multiparous women, respectively without regional analgesia. Indeed, cervical dilatation ranges from at least 1.2 up to 6.8 cm/hr. Cervical dilation in multiparous women is somewhat faster in the active phase, with the minimum rate of 1.5 cm/hr. If the total duration of labor is more than 20 h in nulliparous or more than 14 h in multiparous, it is considered prolonged labor [7].

Cervical dilatation and effacement are the two main factors that determine duration of labor [8]. Several studies showed that active management of labor could shorten the duration of labor, and the safety of this method has been demonstrated [9]. Mechanical, pharmacological and non-pharmacological factors can facilitate cervical dilatation. Prostaglandins, oxytocin, analgesics, and smooth muscle relaxants are examples of pharmacological agents. Several anti-spasmodic agents, including hyoscine n-butylbromide (HBB), drotaverine hydrochloride, phloroglucinol, and valethamate bromide, can shorten the duration of labor [10]. These medications are commonly used during labor in both developing and developed countries [11]. HBB (also known as scopolamine is an anti-cholinergic and anti-muscarinic medication that has shown spasmolytic function on the smooth muscle of the female genital tract, especially the cervico-uterine plexus [12, 13]. This medication can progresses cervical dilatation without any effect on contractions of the uterus [12]. The hyoscine does not pass the brain barrier and does not have a central anti-cholinergic effect. It is rapidly distributed into the tissues after intravenous injection and acts as a cervical spasmolytic agent in labor. The mechanism by which HBB acts in labor is unknown [14]. Several clinical trial studies that describing the effects of HBB on labor progress. Some trials reported that the mean duration of the first stage of labor was significantly shorter in HBB group than that in the placebo group [10, 14–23]. Also, a Cochrane review reported that antispasmodic drugs reduce the duration of the first stage of labor and increased the cervical dilation rate. The antispasmodics drugs used in the Cochrane review included valethamate bromide, hyoscine butyl-bromide, drotaverine hydrochloride, rociverine and camylofin hydrochloride [1]. Other studies that showed conflicting results.

Treveno et al. (2014) and Barau et al. (2018) found no significant differences in the mean duration of active phase of labor between HBB and placebo groups [24, 25].

Given the fact that different studies showed different results, the present systematic review aimed to examine the effect of hyoscine n- butylbromide on the labor progress.

## Methods

This systematic review followed the Systematic Reviews and Meta-Analyses of Randomized Controlled Trials (RCTs). The protocol of this systematic review was registered in the “PROSPERO” with the reference number of CRD42018108703.

### Search strategies

The databases that were searched up to September 2018 included Cochrane Library (Central, 1991), Web of Science (1990), Scoupus, Science Direct (1997), and PubMed (1996). The search was updated up to December 2nd 2019. Some of the search terms used were: Butylscopolammonium Bromide, Bromine, Scopolamine Derviatives, Acceleration of labor, Obstetric, and First Stage of Labor (Supplementary material).

### Inclusion and exclusion criteria

We included the following articles: a) full-text articles published in English or other languages, b) Randomized controlled trials. The studies available only as an abstract were excluded.

### Types of participants

Participants with the following criteria were included; primi- and multigravida women who had a normal singleton term pregnancy (≥ 37 weeks of gestation at the time of delivery; with vertex presentation and intact membrane; spontaneous onset of labor and presence of regular uterine contractions. Also included were women in active phase of labor (cervical dilatation of 3- 4 cm). Women with any contraindication for vaginal delivery and any chronic or pregnancy-induced illnesses were excluded from the study.

### Types of interventions

We included studies where hyoscine n- butylbromide (HBB) was used during the active phase of labour by any route of administration (oral, rectal, intramuscular or intravenous) compared with a placebo.

### Types of outcome measures

#### Primary outcomes

Primary outcomes were as follows

1. Duration of the first stage of labor
2. Duration of the second stage of labor
3. Duration of the third stage of labor
4. Cervical dilation rate
5. Need for augmentation of labor with oxytocin

#### Secondary outcomes

##### Maternal

1. Postpartum hemorrhage rate
2. Blood loss after delivery
3. Caesarean section rate
4. Adverse events

##### Neonatal

1. Apgar score 1 and 5 min after birth
2. Admission to neonatal intensive care unit (NICU)

### Study selection

The due databases were searched, then two authors (ZM) and (AS) independently screened titles and abstracts of search results. The same authors conducted full text screening. All screening, data extraction and quality assessment were done using Covidence software. If there was any disagreement, it was resolved by discussion or getting help from the third author (PA).

### Data extraction

We designed a form according to the data extraction form recommended by the Pregnancy and Childbirth Cochrane Group. Two authors (ZM and SF) independently extracted information on study details including; design of the study, inclusion and exclusion criteria, baseline characteristics, interventions and outcomes. Data extraction was done using Covidence software. Data was entered into Review Manager Software (RevMan 5.3).

### Assessing the risk of bias in included studies

Two reviewing authors (ZM and SF) independently assessed the risk of bias for each study using seven criteria that are required by Cochrane guidelines for quality assessment of randomized controlled trials. The following issues were covered: random sequence generation (selection bias), allocation concealment (selection bias), blinding of the participants and the personnel (performance bias), blinding of outcome assessment (detection bias), incomplete outcome data (attrition bias), selective reporting (reporting bias) and other risk of bias. If there was any disagreement, it was resolved by discussion.

### Statistical analysis

For continuous data such as duration of the first, second and third stage of labor, cervical dilatation rate, Apgar score at one and 5 min after birth, we used the mean and standard deviation with 95% CIs, and for dichotomous data, we presented results as risk ratio or odds ratio (OR) with 95% confidence intervals. All outcomes were measured in the same way between trials. Forest plot was used to demonstrate effect size and CI. Heterogeneity between the included studies was assessed by I^2^. By default, we used fixed effects for all pooled studies. According to the initial results of heterogeneity, if I^2^ > 50%, the random effect model was used. Further, we used sensitivity analysis to discover the potential source of heterogeneity if heterogeneity across studies was statistically significant. Sensitivity analyses were performed by sequentially omitting one single study each time to test the robustness of uncertainty in the meta-analysis. All data were analyzed with Review Manager (RevMan 5.3) statistical software provided by the Cochrane group. The significance level was set at 0.05.

### Subgroup analysis and investigation of heterogeneity

We carried out the following subgroup analyses: primigravida versus multigravida; dosage (20 mg versus 40 mg HBB) and; dosage and gravidity.

## Results

### Literature search

**Fig. 1:**
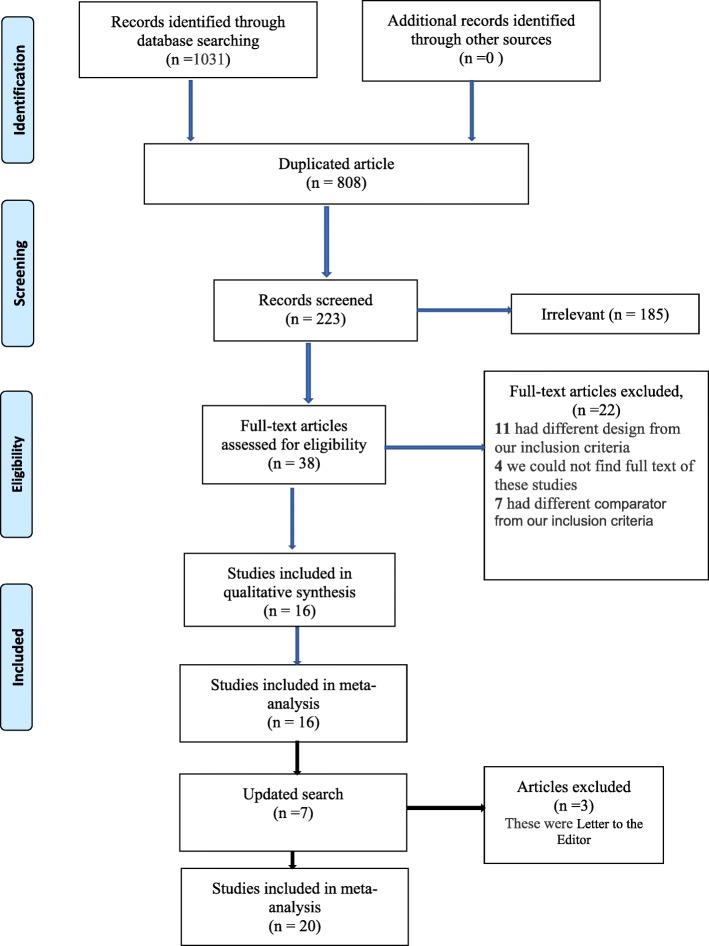
Flow diagram of the study

In the initial search of databases 1031 articles were found. After removing the duplicates (*n* = 808), two reviewers (ZM and AS) screened the titles and abstracts for potentially relevant studies (*n* = 223) independently. Thirty-eight articles were considered as eligible. For four articles, we attempted to contact the authors in order to get the full text versions, but the attempt was not successful, so we excluded these studies [26–29]. Eleven studies were excluded because of study design was different from our inclusion criteria and seven studies were excluded because of their comparators were different from the inclusion criteria of this review. In the search update on December 2nd 2019 four other studies were found [30–33] . Finally, 20 studies were included in the meta-analysis (Fig. 1).

### Characteristics and quality assessment of studies

Twenty studies were included in this meta-analysis. The included studies were published from 2006 to 2019 with RCT design. The characteristics of the included studies are shown in Table 1. Six of the studies were conducted in Iran [19–23, 35]; three in Egypt [16, 18, 36]; three in Nigeria [17, 24, 32]; two in Iraq [30, 31] and one in each following countries: Saudi Arabia [34], West Indies Jamaica [14], Mexico [25], Turkey [10], Puducherry [33] and Pakistan [15]. All studies were performed in the hospital setting, mostly in university hospitals.

**Table 1:** Characteristics of studies included in the systematic review

STUDY	Location	Study type	Age (y) Mean ± SD		No. of participants		Gravidity of participants	Treatment onset	Intervention with dosage	Control with dosage	Duration of first stage of labour Mean ± SD	
			HBB	Placebo	HBB	Placebo					HBB	Placebo
Iravani 2006 [21]	Iran	RCT	**NA**	**NA**	**50**	**50**	**Primigravidas**	**4 cm dilatation and 50% cervical effacement**	**HBB 20 mg (1 ml) intravenously**	**Normal saline 1 ml intravenously**	**166.56** ± **12.85**	**255.98** ± **25.21**
Samuels 2007 [14]	West Indies, Jamaica	RCT	**25.0 ± 6.0**	**26.9 ± 6.2**	**60**	**69**	**Primi- and multigravida**	**4–5 cm dilatation**	**HBB 20 mg (1 ml) intravenously**	**Normal saline 1 ml intravenously**	**156 ± 121.08**	**228 ± 121.08**
Makvand 2010	Iran	RCT	**NA**	**NA**	**65**	**65**	**Primigravidas**	**Established labour,3–4 cm dilatation**	**20 mg Rectal Suppository HBB**	**1 Rectal Suppository placebo**	**141.02 ± 81.86**	**230.09 ± 169.58**
Al Qahtan 2011 [34]	Saudi Arabia	RCT	**23.9 ± 4.04**	**24.4 ± 5.36**	**52**	**45**	**Primigravidas**	**Established labour,3–4 cm dilatation**	**2 mL HBB (40 mg) intramuscularly**	**2 mL normal saline intramuscularly**	**165 ± 67**	**214 ± 79**
Sekhavat 2012 [20]	Iran	RCT	26.1 ± 5.4	26.9 ± 4.8	94	94	**Multigravidas**	**3–4 cm dilatation**	**HBB 20 mg (1 ml) intravenously**	**Normal saline 1 ml intravenously**	186.8 **± 125.6**	**260.4 ± 120.9**
Al-Khishali 2012 [31]	Iraq	RCT	25.9 ± 5.2	25.7 ± 5.5	100	100	**Primi- and multigravida**	**3–4 cm dilatation**	**HBB 20 mg (1 ml) intravenously**	**Normal saline 1 ml intravenously**	167.7 ± 76.2	193.8 ± 58.0
Alani 2012 [30]	Iraq	RCT	27.62 ± 4.179	27.62 ± 3.771	130	130	**Multigravidas**	**4 cm dilatation**	**HBB 40 mg (2 ml) intravenously**	**Normal saline2 ml intravenously**	142.69 ± 44.30	258.00 ± 23.223
Treviño-Salinas 2015 [25]	Mexico	RCT	25.65 ± 6.555	25.37 ± 6.98	43	43	**Primi- and multigravida**	**4 cm dilatation Or more**	**20 mg of BBH (diluted in 9 ml of saline soalution**) **intravenously on two occasions with an interval of 1 h**	**10 ml of saline solution intravenously on two occasions with an interval of 1 h**	**151.186 ± 84.657**	**139.93 ± 92.484**
Kirim 2015 [10]	Turkey	RCT	25.90 ± 6.11	26.18 ± 5.31	197	185	**Primi- and multigravida**	**dilatation of 4 cm and 50% cervical effacement**	**HBB 20 mg (1 ml) intravenously**	**Normal saline 1 ml intravenously**	191.13 **± 43.06**	248.21 **± 66.16**
Edessy 2015 [16]	Egypt	RCT	22.5 ± 1.91	22.3 ± 1.49	44	42	**Primigravidas**	**4–5 cm dilatation**	**HBB 20 mg (1 ml) intravenously**	**Normal saline 1 ml intravenously**	138 **± 37.2**	186 **± 46.8**
Bashir 2016 [15]	Pakistan	RCT	**NA**	**NA**	54	54	**Primi- and multigravida**	**4 cm or more cervical dilation**	**HBB 20 mg (1 ml) intravenously**	**Normal saline 1 ml intravenously**	178.98 **± 92.44**	214.74 **± 147.44**
Shirazi 2016 [19]	Iran	RCT	27(4.56)	27.4(3.94)	30	30	**Primi- and multigravida (until G4)**	**The active phase of labor**	**HBB 40 mg (2 ml) intravenously**	**Normal saline 2 ml intravenously**	426 ± 279	639 ± 237.6
Narappagari 2016 [33]	Puducherry	RCT	24.62 ± 3.53	24.67 ± 3.91	100	100	**Primi- and multigravida**	**Cervical dilatation of 3–4 cm**	**HBB 20 mg (1 ml) intravenously**	**Normal saline 1 ml intravenously**	114 ± 75	182 ± 88
Imaralu 2017 [17]	Nigeria	RCT	28.03 ± 4.79	28.33 ± 5.34	80	80	**Primi- and multigravida (until G4)**	**Cervical dilatation of 4 cm**	**HBB 20 mg (1 ml) intravenously**	**Normal saline 1 ml intravenously**	365.11 ± 37.32	388.46 ± 51.65
Kandil 2017 [18]	Egypt	RCT	23.78 ± 2.65	23.21 ± 2.80	55	55	**Primigravidas**	**The active phase of labor**	**HBB 40 mg (2 ml) intramuscularly**	**Normal saline 2 ml intramuscularly**	208.16 ± 17.24	258.16 ± 15.27
Namaziyan 2017 [35]	Iran	RCT	**NA**	**NA**	150	150	**Primigravidas**	**Dilatation of 4 cm and 30–40% cervical effacement**	**20 mg HBB (1 ml) and 2 cc distilled water intravenously**	**3 cc water distilled intravenously**	405.36 ± 108	350.87 ± 210
Phahlavani-Sheikhi 2017 [22]	Iran	RCT	NA	NA	55	50	**Primigravidas**	**Dilatation of 4 cm**	**HBB 40 mg (2 ml) intravenously**	**Normal saline 2 ml intravenously**	181 ± 59.1	208.2 ± 48.5
Barau 2018 [24]	Nigeria	RCT	29.9 ± 4.9	30.2 ± 4.8	59	64	**Primi- and multigravida**	**Dilatation of 4–5 cm**	**HBB 20 mg (1 ml) intravenously**	**Normal saline 1 ml intravenously**	279.1 ± 134.0	269.3 ± 135.9
Maged 2018 [36]	Egypt	RCT	24.15 ± 3.82	24.43 ± 3.73	40	40	**Primigravidas**	**Dilatation of 3–4 cm and 50% cervical effacement**	**HBB 20 mg (1 ml) + 1 ml saline intravenously**	**Normal saline 2 ml intravenously**	187.73 ± 20.92	231.39 ± 33.14
Ibrahim 2019 [32]	Nigeria	RCT	27 ± 5	25 ± 3	102	102	**Primi- and multigravida**	**Dilatation of 4 cm**	**HBB 40 mg (2 ml) intravenously**	**Normal saline 2 ml intravenously**	344 ± 131	412 ± 175

In all studies, HBB was used in the intervention group and normal saline in the placebo group. HBB was administered either intravenously (IV), intramuscularly (IM) or as a rectal suppository. In 14 studies HBB was administrated with dose of 20 mg [10, 14–17, 20, 21, 23–25, 31, 33, 35, 36], and in six studies HBB was administrated with dose of 40 mg [18, 19, 22, 30, 32, 34]. In two studies, HBB was administrated on two occasions with an interval of 1 h or 4–6 h later [19, 25]. Except for three studies [19, 23, 25], all other studies followed a protocol for active management of labor, which used augmentation with oxytocin, artificial rupture of membranes or both.

Two studies excluded patients from the studies if they underwent augmentation with oxytocin or cesarean section [34, 36]. In one study, patients were routinely given opioid for analgesia after amniotomy (meperidine hydrochloride 100 mg IM and promethazine 25 mg IM) [34].

Eight studies recruited primigravida women [16, 18, 21–23, 34–36]. Ten studies recruited primi- and multigravida women [10, 14, 15, 17, 19, 24, 25, 31–33] and two studies enrolled only multigravida women [20, 30]. In four studies, the duration of the first stage of labor was reported separately by primi and multigravidas [10, 31–33].

The sample size of all studies ranged from 60 to 300. A total of 3108 participants were enrolled of whom 1560 were in HBB group and 1548 in the placebo group.

**Fig. 2:**
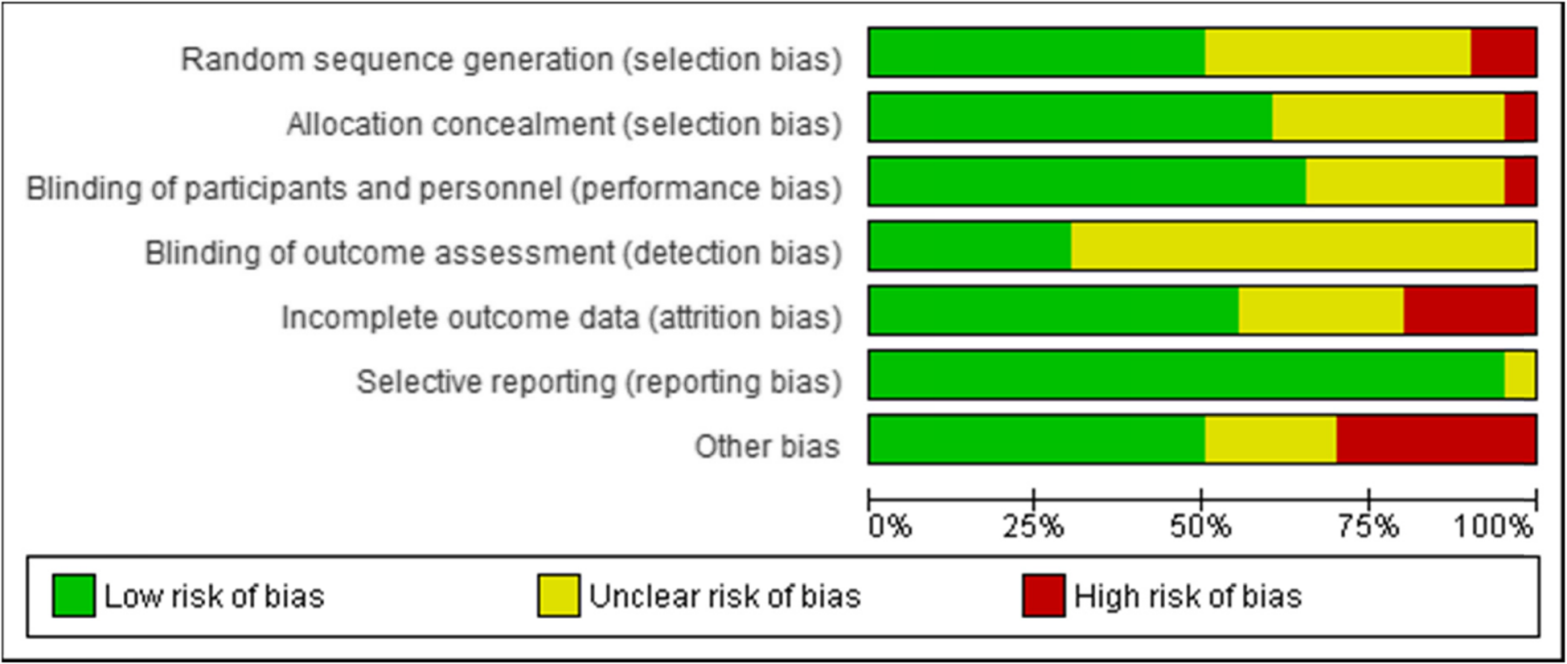
Risk of bias graph: authors’ judgements about each risk of bias item for included studies

The risk of bias for each study was evaluated using the risk of bias assessment for RCTs in Cochrane guidelines. Two individual authors (ZM and SF) evaluated all studies regarding random sequence generation, allocation concealment, blinding of participants and personnel, blinding of outcome assessors, attrition bias, reporting bias and other sources of bias. The risk of bias assessment graph is presented in Fig. 2.

## Overall meta-analysis

### Duration of the first stage of labor

**Fig. 3:**
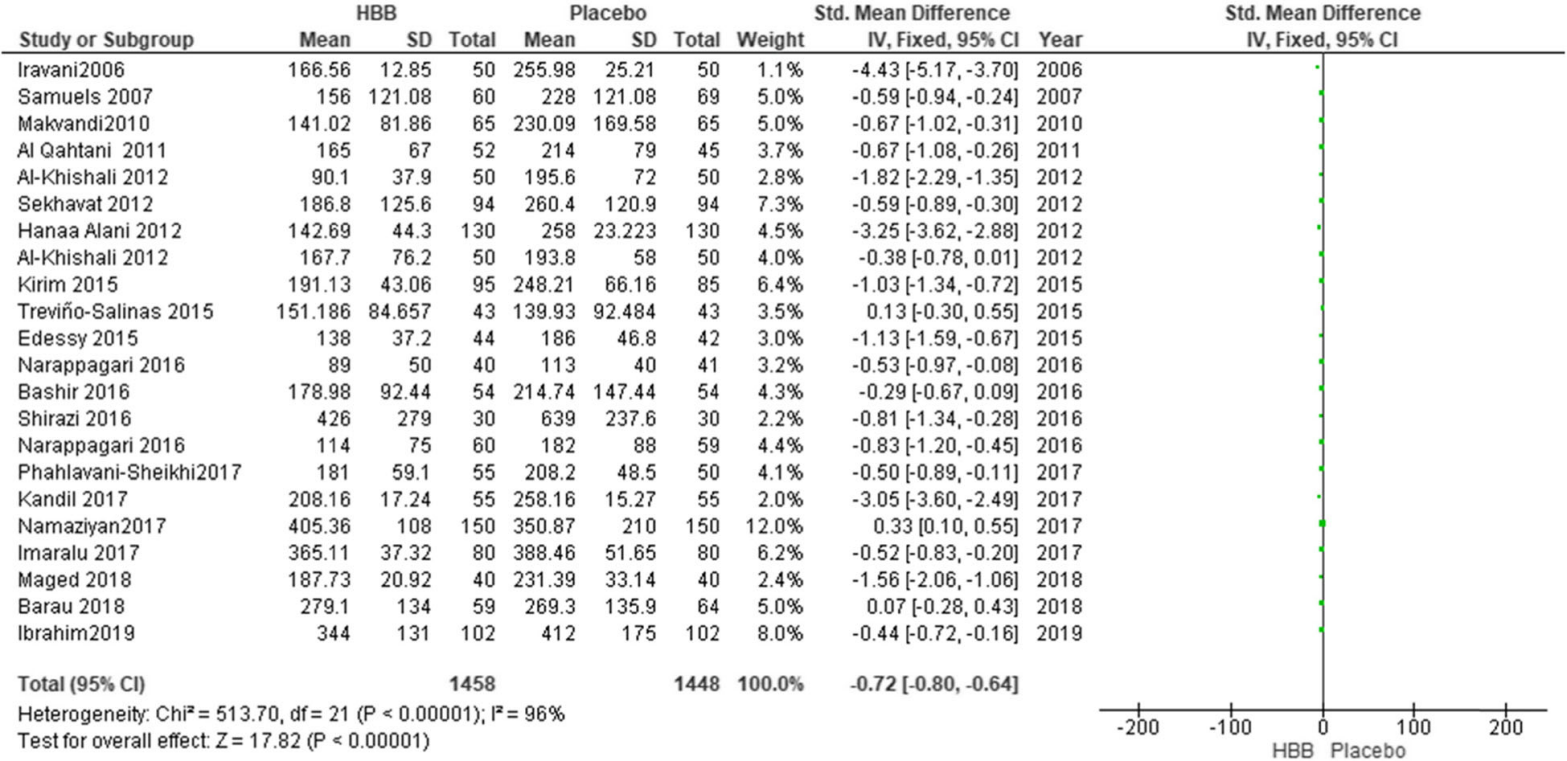
Duration of the first stage of labour in HBB vs placebo group

As indicated in Fig. 3, 3108 women were included in the meta- analysis, of whom, 1560 participants were in HBB group and 1548 in the placebo group. The use of HBB reduced the duration of the first stage of labor by 0.74 min (mean difference − 0.74; 95% confidence interval; − 0.81, − 0.66). While the results indicated a significant difference between HBB and normal saline, a decrease in labor by less than 1 min was found to have no clinically significant effect. Because of the high level of heterogeneity (*P* < 0.00001, I^2^ = 96%), we considered sensitivity analysis and random effect model. Sensitivity analysis was carried out by removing studies one by one to explore the potential source of heterogeneity. By eliminating nine studies, the rate of heterogeneity reduced to 41% (*P* = 0.08) and the statistical difference regarding the effect of HBB on the first stage of labor was not significant anymore.

### Duration of the second stage of labor

**Fig. 4:**
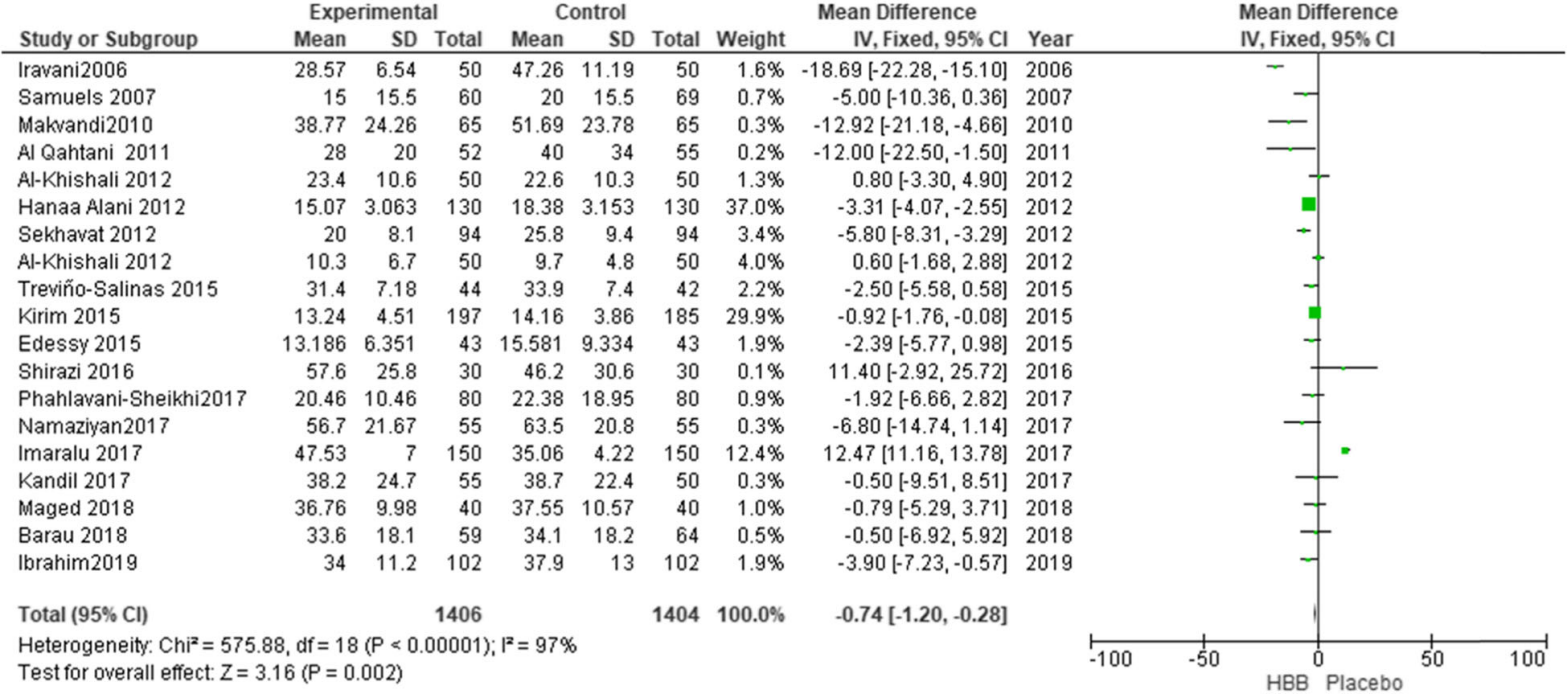
Duration of the second stage of labour in the HBB vs placebo group

Eighteen studies (involving 2795 women) were included in the meta analysis for the duration of the second stage of labor (Fig. 4). There was a decrease in the duration of the second stage of labor by 1.58 min using HBB (MD = − 1.58; 95% CI: [− 2.05, − 1.10]), because this reduction was around 2 min, it has no clinically significant effect.

Because of the high level of heterogeneity (I^2^ = 97%), sensitivity analysis was utilized and by eliminating six studies, the level of heterogeneity was reduced to 40% (MD = − 2.36; 95% CI: [− 3.84, − 0.89]) [10, 16, 17, 21].

### Duration of the third stage of labor

**Fig. 5:**
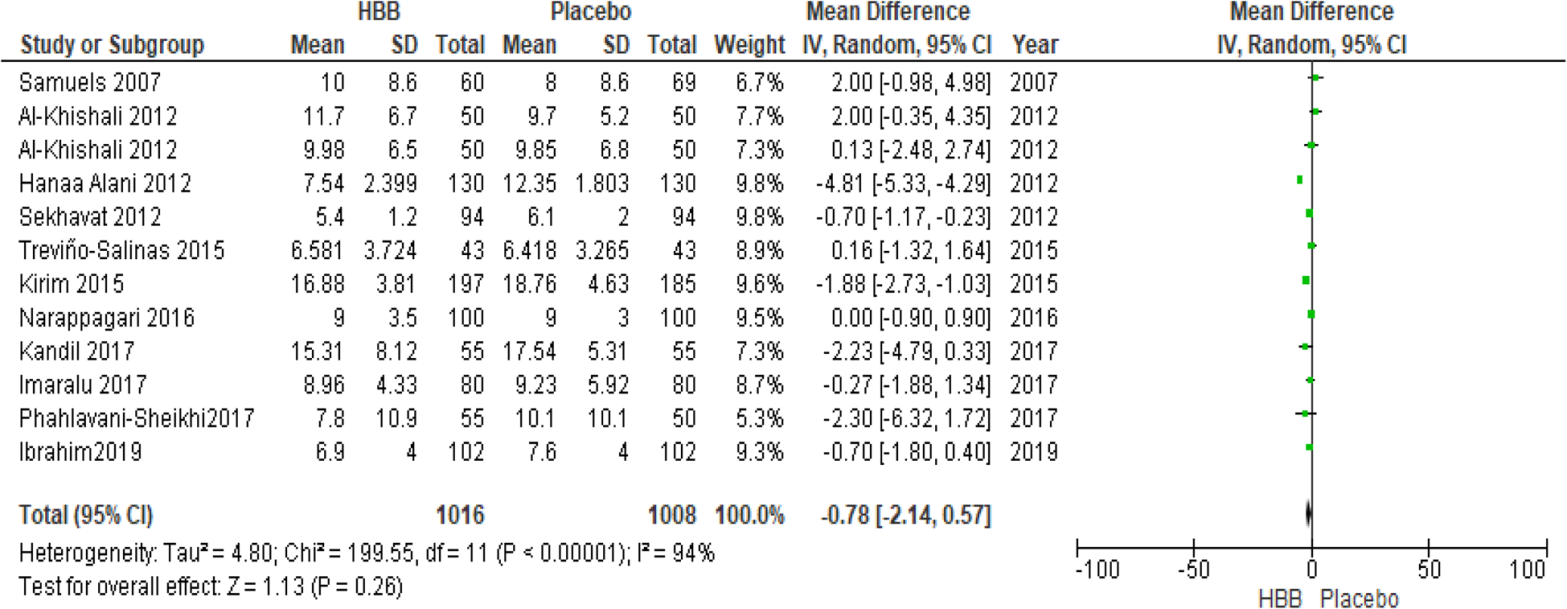
Duration of the third stage of labour in HBB vs. placebo group

Eleven studies (involving 2019 women) were assessed the third stage of labor and were included in the meta- analysis (Fig. 5). HBB could decrease the duration of the third stage of labor by 0.78 min (MD = − 0.78; 95% CI: [− 2.14, 0.57]). Because this reduction was less than 1 min, it has no clinically significant effect.

### Duration of the first + second stages of labor

Four studies (involving 404 women) assessed the duration of the first + second stages of labor [14, 19, 24, 34]. There was an average reduction of 54.66 min in the duration of first + second stages of labor (MD = − 54.66; 95% CI: [− 78.44, − 30.88], I^2^ = 66%). Because of the high level of heterogeneity, sensitivity analysis was used and by removing Barau’s study (2018), the level of heterogeneity was reduced, but the difference between the groups regarding the total duration of the labor did not remain significant (MD = − 70.39; 95% CI: [− 97.04, − 43.73], *P* = 0.33; I^2^ = 11%).

#### Sub-group analysis by gravidity

**Fig. 6:**
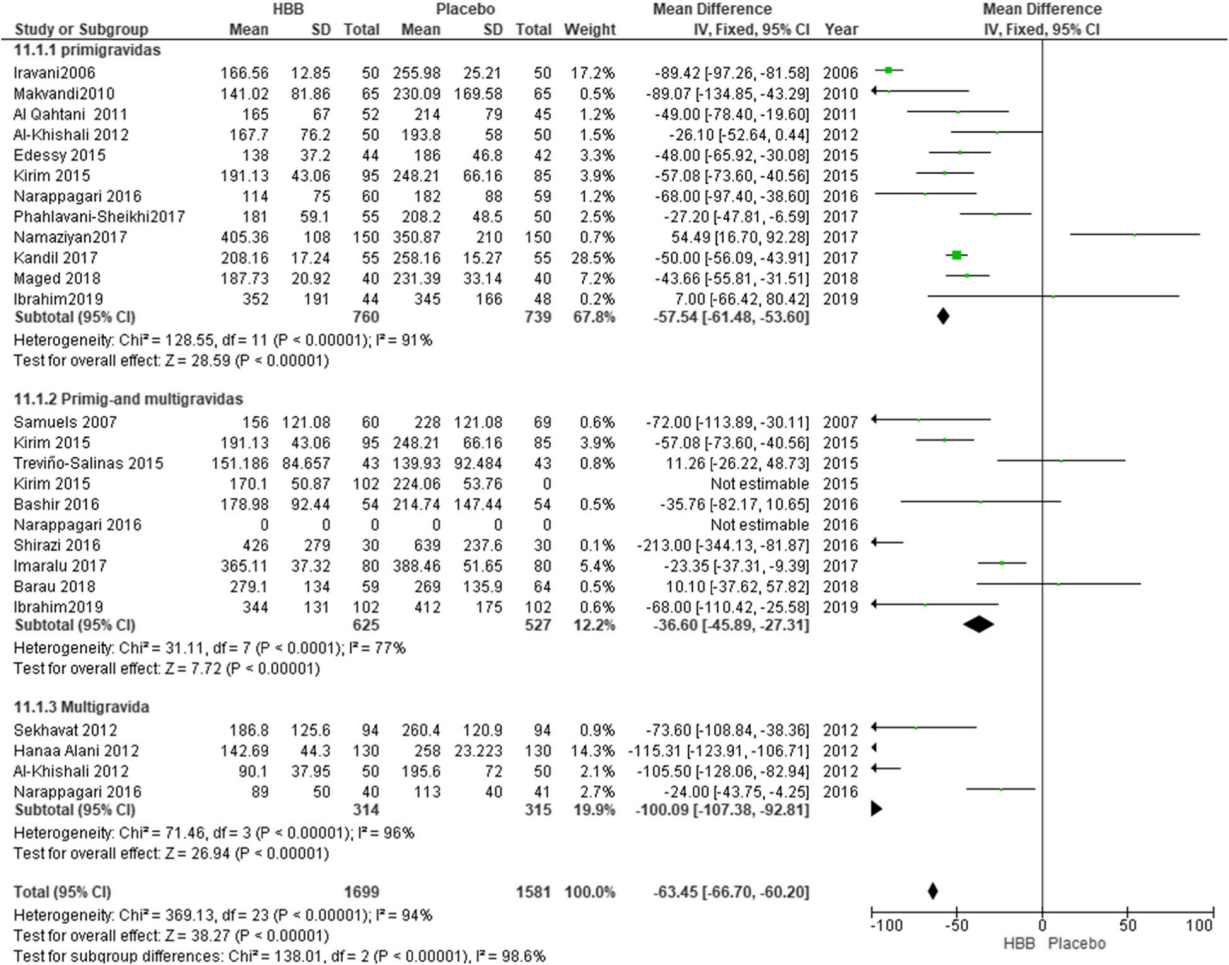
Duration of the first stage of labour in primigravida versus primi-and multigravidas’ and multigravida women

All twenty studies, including 3180 participants, were sub-grouped by the gravidity of the participants. Eleven studies included 1407 primigravida women, five studies included 831 multigravida women and seven studies involved 870 primi-multigravida women. Overall the duration of the first stage of labor was decreased by 63.36 min (MD = − 63.36; 95% CI: [− 66.59, − 60.12]). Among primigravida participants, there was a decrease by 57.73 min in the first stage of labor (MD = − 57.54; 95% CI: [− 61.68, − 53.78]), (Fig. 6). Multigravida women, as seen in Fig. 6, had the highest reduction in the first stage of labor (MD = − 90.74; 95% CI: [− 97.24, − 84.24]). Because of the high level of heterogeneity (I^2^ = 91%), sensitivity analysis was used and by eliminating of nine studies the level of heterogeneity was reduced to 44% (MD = − 47.80; 95% CI: [− 52.24, − 43.57], *P* = 0.04) [17, 19, 21, 23, 24, 30, 35]. In studies which did not distinguish between multigravida and primigravida, the effect was less than those only involving primi- or multigravida women. The duration of labor in these studies decreased by 27.13 min (MD = − 27.13; 95% CI: [− 38.36, − 15.90]).

**Fig. 7:**
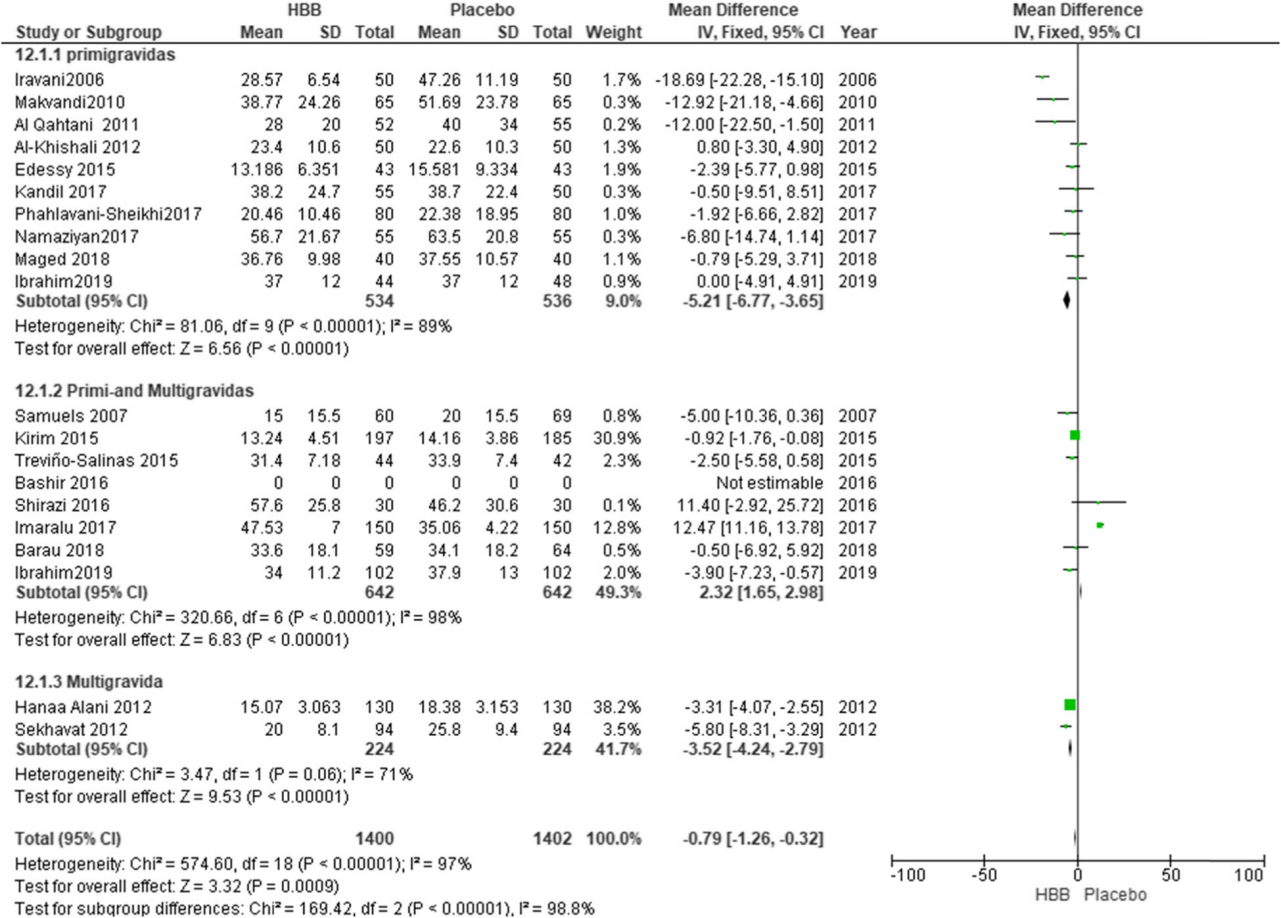
Duration of the second stage of labour in primigravidas versus primi-and multigravida and multigravida women

Subgroup analysis for the duration of the second stage of the labor showed that the use of HBB reduced the duration of this stage of labor between primigravidas vs. primi-and multigravidas, and multigravidas, but had no clinical significant effect (MD = -1.58; 95% CI: [− 2.05, − 01.10]) (Fig. 7). Furthermore, in primipara women, the duration of this stage was reduced by 6.02 min (MD = -6.02; 95% CI: [− 7.64, − 4.40]).

**Fig. 8:**
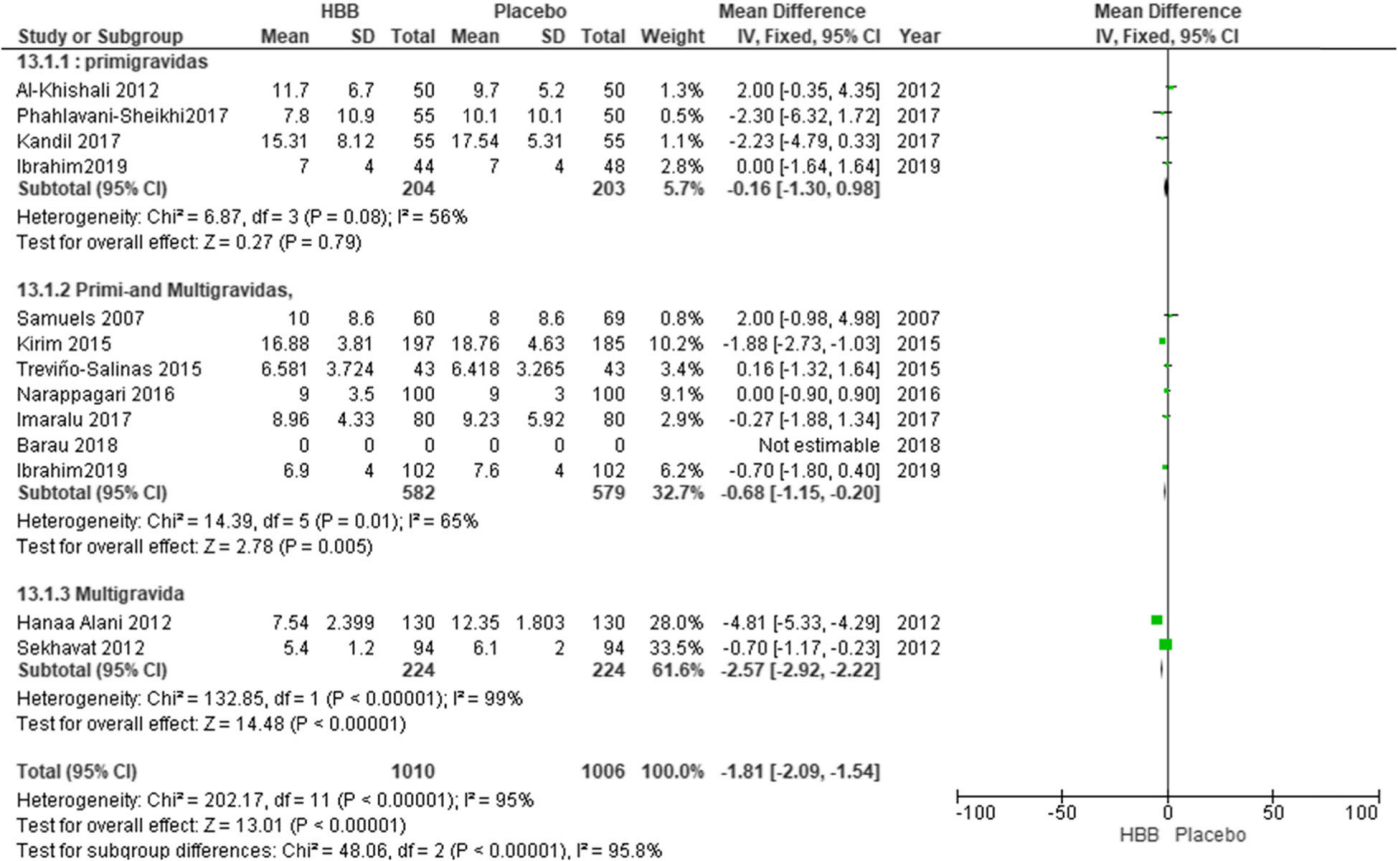
Duration of the third stage of labour in primigravidas versus primi-and multigravidas and multigravida women

Also, subgroup analysis of the duration of the third stage of labor showed significant differences between primigravidas vs. primi-and multigravidas and multigravidas (MD = − 1.84; 95% CI: [− 2.12, − 1.57]) (Fig. 8)). Because of the high level of heterogeneity (I^2^ = 59%), sensitivity analysis was used, and by eliminating of two studies, the level of heterogeneity was reduced to 22% (MD = − 0.43; 95% CI: [− 0.78, − 0.09], *P* = 0.23) [10, 30].

#### Sub-group analysis by dosage

Subgroup analysis was performed for 20 studies containing 3108 participants based on 20 mg and 40 mg HBB dosage. Fourteen studies, including 2272 women, were in the 20 mg HBB dosage subgroup, while six studies including 836 women were in the 40 mg HBB dosage subgroup. Overall, the first stage of labor decreased by 64.74 min (MD: − 64.74, 95% [− 67.97, − 61.50]). Because of the high level of heterogeneity (I^2^ = 94%), sensitivity analysis was used and by eliminating of twelve studies the level of heterogeneity was reduced to 43% (MD = − 49.23; 95% CI: [− 55.97, − 42.48]) [10, 15, 17–19, 21, 24, 25, 30, 31, 33, 35]. A dosage of 20 mg HBB decreased labor by 61.01 min (MD: − 61.01, 95% [− 65.44, − 56.58]). A dosage of 40 mg HBB decreased the duration of the first stage of labor by 68.99 min (MD: − 68.99, 95% [− 73.73, − 64.26]).

### Other outcomes

Six studies (involving 986 women) investigated cervical dilatation [10, 16, 18, 20, 24, 34]. Results of meta-analysis showed a significant increase in the rate of cervical dilatation (MD = 0.21; 95% CI: [0.18, 0.24]). Because of the high level of heterogeneity (I^2^ = 94%), sensitivity analysis was used. By eliminating four studies, two studies (involving 505 women) remained and showed no significant effect of HBB on the rate of cervical dilatation (MD = 0.12; 95% CI: [0.05, 0.19], *P* = 0.19; I^2^ = 42%).

Four studies (involving 840 women) were included in the meta- analysis dealing with the effect of HBB in case of labor augmentation with oxytocin [10, 17, 18, 20]. There was no significant difference in performing augmentation of labor with oxytocin between intervention and control groups (OR: 0.85; 95% CI: [0.64, 1.13]).

Nine studies (involving 1430 women) were included in the meta-analysis for assessing the rate of caesarean section [14, 16, 18, 23, 30–34]. HBB had no significant effect on the rate of caesarean section (OR = 0.77; 95% CI: [0.52, 1.16]).

Four studies with 640 participants reported postpartum hemorrhage rate [17, 18, 22, 30]. However, only the study by Imaralu et al. reported the actual frequency of PPH (OR: 0.11; 95% CI: 0.01, 0.93). Two studies with 289 women reported the amount of blood loss after delivery [14, 17]. There was more blood loss in the placebo group by 9.01 mL) MD: 9.01, 95% [− 46.23, 64.24]).

Maternal adverse events with HBB reported across studies were as follows: tachycardia, mouth dryness, headache, nausea, vomiting, dizziness, cervical laceration, face flushing and postpartum hemorrhage. Meta-analysis was conducted for tachycardia, mouth dryness and blood loss. Six studies involving 1054 women were included in the meta-analysis for the effect of HBB on mouth dryness (OR: 2.15; 95% CI: [1.51, 3.07]) [17, 18, 31, 32, 35, 36]. Because of the high level of heterogeneity (I^2^ = 71%), sensitivity analysis was utilized and by eliminating.

one study, the level of heterogeneity was reduced to 0% (MD = 1.25; 95% CI: 0.84, 1.87]), but the difference was not significant [35].

Six studies involving 910 women were included in the meta-analysis for the adverse effect of tachycardia [17–19, 31, 35, 36]. There was an increased relative risk for tachycardia for participants receiving HBB (OR: 6.10; 95% CI: [3.01, 12.35]). Because of high level of heterogeneity (I^2^ = 66%), a sensitivity analysis was utilized, and by eliminating two studies, the level of heterogeneity was reduced to 0% (MD = 1.42; 95% CI: 0.55, 3.68]), but the difference was not significant.

Neonatal adverse events were reported using different approaches including admission to neonatal intensive care unit (NICU), fetal tachycardia, need to resuscitation and Apgar score one and 5 min after delivery.

Five studies involving 703 babies were included in the meta-analysis for assessment of admission to NICU [16, 18, 20, 22, 31]. There was no significant difference between HBB and placebo in the ratio of admission to NICU (OR: 0.99; 95% CI: [0.35, 2.75]). In 11 studies involving 1682 participants, there was no significant difference in the Apgar score at 1 min between HBB and control groups (MD = - 0.04; 95% CI: [− 0.09, − 0.01]).

[10, 14, 16–18, 20, 21, 23–25, 30, 36]. Meta-analysis of Apgar score at 5 min after birth in 12 studies showed a significant difference between HBB and control groups (MD = - 0.05; 95% CI: [− 0.10, − 0.00]) [11, 15, 17–19, 21, 22, 24–26, 32, 30)]. Because of the high level of heterogeneity, sensitivity analysis was used and after eliminating foure studies, results of seven studies (involving 1453 babies) showed no significant effect of HBB on APGAR score at 5 min after birth (MD = − 0.03; 95% CI: [− 0.08, 0.03]) [14, 16, 18, 24, 25].

## Discussion

This systematic review aimed to investigate the effect of hyoscine on the progression of labor. In this systematic review, 20 studies were included. The studies mostly have been conducted in Iran or low or middle-income countries. Hyoscine is a drug belonging to the anti-cholinergic anti-spasmodic group, which exerts its relaxing effects directly on the smooth muscles of the digestive as well as urogenital systems [23].

Studies conducted on the use of HBB to shorten the active stage of labor showed conflicting results. Overall, HBB shorten labor by almost an hour at both 20 mg and 40 mg doses in primigravida and multigravida women.

Subgroup analysis between primigravida, multigravida and prim- multi-gravida indicated that the duration of the first stage of labor was decreased by 63.45 min. Multigravida women had the highest reduction in the first stage of labor by 90.74 min, and this stage of labor in primigravida participants was decreased by 57.73 min. In Kirim 2015 [11], Al-khishali 2012 [32], Narappagari 2016 [35] and Alani 2012 [31], the duration of the first stage of labor was reported per parities, and we considered this in analysis of the subgroups. Since the rate of labor progression is different between multigravida and nulliparous women, this can affect the overall results of the investigation. In this regard, in subgroup analysis, we observed that use of HBB decreases the duration of the first stage of labor in primigravida women, in comparison to primi- multigravida and multigravida by 57.73, 27.13 and 90.74 min respectively. Further, the total duration of labor showed a significant difference between the three groups (*p* = 0.00001). However, use of HBB could reduce the total duration of the first stage of labor about 63.36 min, and after sensitivity analysis the significance changed to 47.80 min.

The results of the study by Rohwer et al. [1] on the effect of anti-spasmodic agents including hyoscine, Valethamate bromine, and durtavirine on labor including 17 studies in the meta-analysis showed that overall, the use of these medications significantly decrease the duration of labor by 73.34 min. The discrepancy between Rohwer et al. and the present study may be because the distribution of parity includes 1407 primipara women, 870 primi-multigravida and 831 multigravidas and type of the interventions performed were different among studies. Also, six anti-spasmodic agents were used for the labor improvement among studies in the Rohwer et al’s study. Ranjbaran et al., in a review study found that HBB could significantly reduce the first and the second stages of labor, but not the third stage of labor. Their review included 10 studies that published in the English language [37].

Measurement of the duration of labor can be challenging and subjective because of the following reasons. First, differences in measuring the cervical dilatation rate can be potentially affected by detection bias. Secondly, due to individual differences in women, the labor progress varies considerably between women [1]. Thus, in most studies, the first stage of labor is considered as the starting point of interventions. In the present study, the time of administration of hyoscine was at 3–4 cm dilatation with effacement of 50% or more. Meta-analysis of six studies indicated that cervical dilatation had increased significantly with using HBB. In investigation of subgroups, hyoscine was useful for cervical dilatation in primgravida women as 0.21 cm/h and 0.12 cm/h for primi-multigravida women. In Rohwer study, the administration of antispasmodic agents significantly increased the rate of cervical dilatation by an average of 0.61 cm/hour (MD 0.61 cm/hour; 95% CI: 0.34 to 0.88) [1].

In the present systematic review, HBB could significantly decrease the duration of the second stage of labor across all studies by 1.58 min. Further, in investigation of subgroups, the duration of this stage decreased significantly in primi gravida women by 5.80 min.

The duration of the third stage of labor in eleven studies in the HBB group was reduced by 0.78 min. Nevertheless, the difference between the two groups did not remain significant after sensitivity analysis. Further, in the analysis of subgroups, the duration of this stage decreased significantly by 1.79 min.

The aim of the active management of labor is shortening the duration of labor without increasing maternal and neonatal risks [10]. If the uterus does not have a proper function and is hypotonic, i.e. the number of contractions is less than three times per 10 min, or the duration of contraction is less than 40 s, amniotomy and induction with oxytocin are recommended for active management of labor. In the present meta-analysis four studies which used amniotomy and oxytocin induction in cases with lack of labor progression indicated that the use of amniotomy and oxytocin augmentation did not differ significantly between the two groups.

The results of nine studies in this meta-analysis revealed that use of HBB reduces the rate of cesarean, but the difference was not significant.

Adverse maternal and neonatal outcomes using HBB were not reported completely in all studies. Mouth dryness and tachycardia were among the conditions reported in most studies. In six studies, use of hyoscine significantly increased the risk of mouth dryness, and in six studies, it increased the risk of tachycardia significantly. Differences about mouth dryness and tachycardia disappeared after sensitivity analysis. Regarding neonatal adverse effects, most studies have reported APGAR score in one and 5 min after birth, and there was no significant differences between the two groups. Another neonatal adverse event that was reported in five studies was the rate of admission in the neonatal intensive care unit, which was not statistically different between the two groups.

Only in the study by Makvandi et al., the effect of hyoscine had been examined on labour pain, which indicated that hyoscine had no effect of the intensity of labor pain [23].

It is believed that shortening the duration of the first stage of labor has many potential benefits including diminished chorioamnionitis, neonatal sepsis, post-labor sepsis, and the decreased demand for using repeated doses of opioids and sedatives, which are associated with neonatal respiratory depression. Also, the use of safe interventions that result in a shortened duration of labor and pain could significantly increase the sense of satisfaction in mothers [14]. Most studies of this meta-analysis have been performed in low- to middle-income countries. In Iran, according to the national guidelines, the provision of midwifery and obstetric services include the use of adjuvant therapies of labor analgesia including opioids and sedatives. However, hyoscine is not one of the drugs recommended in the national protocol [38]. Nevertheless, it is used in some hospitals to shorten the duration of labor. In India, although the use of anti-spasmodic agents are recommended in the the national protocol, it seems that use of these drugs especially their safety is still under debate [14].

### Quality of studies

Random sequence generation was high-risk, unknown, and low-risk in two, eight, and ten studies, respectively. All studies except for seven specified the allocation concealment. Many studies were judged as unclear risk of bias, especially for detection bias, as 14 studies did not discuss whether their outcome assessors were blinded. Only one study did not blind participants and personnel and juged as a high risk of performance bias. Nine studies did not determine whether they were sponsored by a drug company or had any conflicts of interests.

### Limitations of this study

Several limitations existed in this systematic review: 1) labor duration in studies in which the participants were primi-and multigravida was not presented based on separate parities except for four paper; 2) the route of HBB administration was not the same across all studies; 3) the maternal and neonatal outcomes have not reported completely; 4) in one study, in addition to HBB, opioids have also been used. These limitations could have contributed to heterogeneity.

## Conclusion

Although, the effect of HBB was minimal when multigravidas and primigravidas women were considered together, the HBB was clinically effective in primigravida and multigravida women for shortening the first and the second stages of labor. The effect of HBB on shortening the duration of the third stage of labor was significant only in multigravidas. Adverse maternal and neonatal outcomes are negligible, especially at the 20 mg HBB dose.

Considering the limited resources of low or middle income countries, administering of HBB may be recommended for shortening labor.

### Implications for research

A truly rigorous RCT must still be conducted on this topic, especially controlling for augmentation of labor with other factors like oxytocin. Also, since many studies did not report the adverse maternal or neonatal events, future research should be conducted to determine the safety of HBB during labor.

## Supplementary information

**Additional file 1.**

## Data Availability

Not Applicable.

## Abbreviations

HBB: Hyoscine n-butylbromide
MD: Mean difference
NICU: neonatal intensive care unit
RCT: Randomized controlled trial
