# Supplementary material for: The effect of hyoscine n- butylbromide on labor progress: A systematic review
Source: BMC Pregnancy Childbirth. 2020 May 13;20:291. doi: 10.1186/s12884-020-2832-3 (PMC7218842; doi:10.1186/s12884-020-2832-3)
Supplement: Supplementary file 1 — Additional file 1. [file 12884_2020_2832_MOESM1_ESM.docx]

This message contains search results from the National Center for Biotechnology Information ([NCBI](http://www.ncbi.nlm.nih.gov/)) at the U.S. National Library of Medicine ([NLM](http://www.nlm.nih.gov/)). Do not reply directly to this message

Sent on: Wed May 23 03:16:15 2018

Search: ((((((((Butylscopolammonium Bromide OR hyoscine n butylbromide OR Bromine OR Scopolamine OR Scopolamine Derivatives OR Buscapine OR Buscolysin OR Buscopan OR Butylscopolamine OR Hyoscinbutylbromide OR Hyoscine N-Butylbromide OR N-Butylscopolammonium Bromide OR Scopolaminebutylbromide OR Scopolan[MeSH Terms])) OR (Butylscopolammonium Bromide[Title/Abstract] OR hyoscine n butylbromide[Title/Abstract] OR Bromine[Title/Abstract] OR Scopolamine[Title/Abstract] OR Scopolamine Derivatives[Title/Abstract] OR Buscapine[Title/Abstract] OR Buscolysin[Title/Abstract] OR Buscopan[Title/Abstract] OR Butylscopolamine[Title/Abstract] OR Hyoscinbutylbromide[Title/Abstract] OR Hyoscine N-Butylbromide[Title/Abstract] OR N-Butylscopolammonium Bromide[Title/Abstract] OR Scopolaminebutylbromide[Title/Abstract] OR Scopolan[Title/Abstract]))) AND (((Acceleration of labor OR Accelerated delivery OR First Stage of Labor OR Labor, Obstetric OR augmentation[MeSH Terms])) OR (Acceleration of labor[Title/Abstract] OR Accelerated delivery[Title/Abstract] OR Labor, Obstetric[Title/Abstract] OR First Stage of Labor[Title/Abstract] OR augmentation[Title/Abstract])))
